# Improved Formability of Mg-AZ80 Alloy under a High Strain Rate in Expanding-Ring Experiments

**DOI:** 10.3390/ma11020329

**Published:** 2018-02-24

**Authors:** Shmuel Samuha, Eyal Kahana, Oren Sadot, Roni Z. Shneck

**Affiliations:** 1 Department Materials Engineering, NRCN, P.O. Box 9001, Beer-Sheva 84190, Israel; eyal8365@gmail.com; 2 Israel Atomic Energy Commission, P.O. Box 7061, Tel-Aviv 61070, Israel; 3 Department Mechanical Engineering, Ben-Gurion University of the Negev, Beer-Sheva 84105, Israel; sorens@bgu.ac.il; 4 Department Materials Engineering, Ben-Gurion University of the Negev, Beer-Sheva 84105, Israel; roni@bgu.ac.il

**Keywords:** magnesium alloy, forming, deformation, recrystallization, electron back-scattered diffraction, twinning, texture, slip

## Abstract

Magnesium alloys offer a favored alternative to steels and aluminum alloys due to their low density and relatively high specific strength. Their application potentials are, however, impeded by poor formability at room temperature. In the current work, improved formability for the commercial magnesium AZ80 alloy was attained through the application of the high-rate electro-magnetic forming (EMF) technique. With the EMF system, elongation of 0.2 was achieved while only 0.11 is obtained through quasistatic loading. Systematic microstructural and textural investigations prior, during and post deformation under high strain-rate experiments were carried out using electron back-scattered diffraction (EBSD) and other microscopic techniques. The analysis indicates that enhanced elongation is achieved as a result of the combination of deformation, comprising basal and non-basal slip systems, twinning and dynamic recrystallization. An adopted EMF-forming technique is tested which results in enhanced elongation without failure and a higher degree of dynamically annealed microstructure.

## 1. Introduction

Magnesium (Mg) alloys have the potential to reduce fuel consumption in the automotive and aviation industries due to their low density (1.74 gm/cm^3^) and high specific strength-to-weight ratio. However, the ductility of magnesium alloys is limited due to their hexagonal close pack (HCP) structure, making conventional forming methods at room temperature almost impossible [1,2]. Therefore, most magnesium alloys for automotive parts are cast alloys manufactured by die-casting techniques [3]. 

The activity of the different deformation modes is mainly determined by its critical resolved shear stress (CRSS) which largely depends on the energy and mobility of its dislocations and is thus affected by the experimental temperature, strain rate, texture, *c*/*a*-ratio, and so forth [4]. At room temperature, an insufficient number of slip systems are activated, thus plastic deformation is also accommodated by twinning [5,6]. The prevailing slip is in the close-packed <*a*> directions on the {0002} basal and the {$10\bar{1}0$} prismatic planes. Additional systems (pyramidal <*c* + *a*> {$11\bar{2}2$} <$11\bar{2}\bar{3}$> and {$10\bar{1}1$} <$11\bar{2}0$>) are also active, albeit to a lesser extent [4]. It is generally accepted that at a low strain rate and low temperature, the CRSS for the basal glide is smaller than that of twinning [5,7]. On the other hand, for the same <*a*> glide, but on the {$10\bar{1}0$} prismatic planes, high stresses are required. In contrast to basal glide and twinning, the prismatic and the pyramidal systems are temperature dependent [8]. At an elevated temperature, their CRSS is strongly reduced, even to smaller levels than the CRSS of the twinning [9,10]. 

The crystallography of the twins in hexagonal symmetry materials and their impact on the lattice re-orientation have been thoroughly documented in the literature ([11,12] for example). The deformation twins are nucleated, with the most commonly observed twins being the {$10\bar{1}2$} extension and the {$10\bar{1}0$} contraction twins. Twinning is a polar mechanism [13] as the type of the nucleated twin is dependent on the texture with respect to the loading direction. The extension twins along the {$10\bar{1}2$} take place if the direction of tensile loading is parallel to the *c*-axis and when a compressive load is perpendicular to the *c*-axis is applied. In turn, contraction twins along the {$10\bar{1}0$} take place when a compressive load along the *c*-axis is applied or if the direction of tensile loading is perpendicular to the *c*-axis [14,15,16,17]. Due to the complicated interaction between twinning and slip, twining plays a twofold role in the formability of HCP metals. On the one hand, twin boundaries act as effective barriers to the dislocation motion (hardening) [18] and, on the other hand, twins present preferred nucleation sites for recrystallized grains in the plasticly deformed microstructure (softening) [19,20]. The contribution of twins in the accommodation of plastic deformation is also strain-rate dependent. Their volume fraction increases substantially in a high strain-rate deformation at low temperature ([21,22], for example). As mentioned earlier, twinning is known to be a thermal mode of deformation. Thus, at an elevated temperature under low strain rates, twinning generally becomes less prevalent and deformation is predominantly by crystallographic slip [4,9,10]. 

The improved formability of Mg alloys at room temperature can be obtained by utilizing the electromagnetic forming (EMF) technique [23]. EMF processes operate by discharging stored energy from a capacitor bank into a coil. The coil transforms electrical energy into magnetic energy due to the rapid change in current over a very short period of time. The magnetic field induces eddy currents in the workpiece that oppose the primary magnetic field and create repulsive forces. The magnetic force accelerates the workpiece to a very high velocity, giving rise to high strains (10^−3^ to 10^−4^ s^−1^) in extremely short times (μs). A common way of studying the EMF formability of metals and alloys is the expanding ring method using an internal copper coil. Much research has been conducted on the applicability of EMF to metals with cubic symmetry, such as aluminum and copper rings [24,25,26,27]. On these face-centered cubic (FCC) materials, expansion-ring tests result in approximately double the ring ductility with the accessible strain rates [24]. Moreover, the total strain-to-failure increased with an increase in the strain rate. This enhanced ductility was attributed to their FCC structures, which provides isotropic plastic strain by its 12 equivalent slip systems. For hexagonal symmetry, to the best of our knowledge, the EMF technique has only been applied on AZ31 [28], where it was reported to increase the strain before failure up to 0.34 (2.5-fold higher than reported for quasistatic tensile tests).

In the current study, the high strain-rate deformation of the Mg-AZ80 alloy is explored, employing the EMF technique. The deformation mechanisms are systematically studied by microstructure characterization and texture analysis during and immediately after high strain-rate expansion tests.

## 2. Materials and Methods

### 2.1. Material and Test Specimens

The starting material was a commercial AZ80 (8.66 wt % Al, 0.40 wt % Zn, 0.17 wt % Mn, Mg–balance) extruded bars, machined into rings. The outer diameter of the rings was 36.0 mm, the inner diameter was 32.6 mm and the ring height was 2.4 mm. The rings were homogenized at 410 °C for 18 h in an argon protective atmosphere, followed by air quenching.

Macro and microstructure characterization as well as texture analysis were performed on three specimens. The initial state is described based on the characterization of a ring prior to the EMF experiment. Fragments from an expanded ring which drop into water immediately after the test are referred as the ‘as-quenched’ condition. Fragments that were cooled in free air are referred to as the ‘air-cooled’ specimen.

### 2.2. Electromagnetic Forming (EMF) System and Analysis

**Scheme 1:**
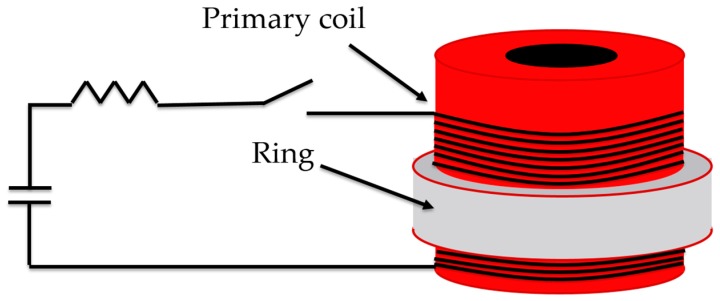
Diagram of the electromagnetic forming (EMF) system.

The EMF system was equipped with a 200 µF capacitor, loaded to high voltage, then discharged through a resistor–inductor–capacitor (R-L-C) circuit. The discharge current flows within the coil that is placed inside the ring workpiece, and a corresponding opposite directed current is induced in the workpiece so that the energy density of the magnetic field strength in the gap between tool coil and workpiece can cause ring deformation [29]. Details on the electric system and ring expansion can be found in [30,31]. A schematic presentation of the EMF system is presented in Scheme 1. 

The temperature of the rings could not be measured directly due to the safety precautions that allow entry into the experimental area only 60 s after the electric capacitor discharged. After this period, the temperature of the fragments was slightly higher than room temperature, comfortable to the touch. 

In EMF experiments, different strain rates and strain-to-failure are obtained employing different acceleration voltages. Using a technique described in [29], these results were documented. Through trial-and-error experiments, it was found that for a 4.6 kV discharge, a maximum strain-to-failure of 0.2 ± 0.01 is reached when the strain rate higher than of 6.7 × 10^−3^ s^−1^. These operational conditions were used throughout all expansion experiments presented in this work.

### 2.3. Sample Preparation

General observation of the rings was made using optical microscopy (OM). A sample preparation for OM included mechanical grounding with SiC papers of grit sizes down to 4000. For scanning electron microscope–electron backscatter diffraction (SEM–EBSD) analysis, mechanical grounding was followed by chemical polishing (rinse in a Nital solution composed of 5% nitric acid and 95% absolute ethanol for 60 s at room temperature). Then, the samples were quickly rinsed with ethanol and dried under a blast of air. For transmission electron microscope (TEM) characterization, samples were sectioned using electric-discharge machining (EDM). Then, sections were paired (face-to-face) using GATAN two-component epoxy glue of mixed hardener and resin in a ratio of 1:10, respectively. The TEM sample was mechanically ground with SiC papers of grit sizes down to 4000. A supporting Cu ring was glued on top of the first polished side of the specimen. Transparency was achieved through Ar–ion bombarding using an ion-milling machine.

### 2.4. Crystallographic Characterization Protocols

Analysis of the deformed microstructure was undertaken throughout the procedure using a TEM operating at 200 kV and equipped with a slow scan camera. TEM images were constructed using conventional forward-scattered (bright-field (BF)) or diffracted beams (dark-field (DF)). Defects, such as dislocation structures and twins, were emphasized using near weak-beam dark-field (WBDF) illumination conditions. Microstructure and texture analysis was carried out using SEM–EBSD within a SEM with a field-emission gun operated at 20 kV. The principal directions of a SEM–EBSD sample are denoted by the extrusion direction (ED), the transverse direction (TD), and the normal direction (ND). The SEM–EBSD microstructure characterization was carried out on the ND–TD plane, from a region of interest (ROI) of 500 × 500 μm. A step size of 0.5–1 µm was chosen, depending on the grain size and successful interpretation of the Kikuchi patterns (severity of local deformation). During each EBSD data collection, more than 243,000 points were recorded, with hit rates of 94–99%. The MTEX package, a freely available MATLAB toolbox, was employed for post-processing and visualization of EBSD data [32]. A grain-boundary character distribution (minimum rotation angle between neighboring points of measurement) was determined based on a misorientation angle criteria—in the range of 2–15° for low-angle grain boundaries (LAGBs) and higher than 15° for high-angle grain boundaries (HAGBs). Grain-size measurements were made based on the EBSD maps by the grain-reconstruction method [33]. In this approach, the grain’s area is measured, and the equivalent circular diameter (ECD) grain is calculated. Figures on the grain size and morphology are referred in the present paper as “grain-structure maps”. Qualitative intra-grain strain analysis and recrystallization fraction was performed based on the local misorientation approach using kernel-average misorientation (KAM) maps and the grain-orientation spread (GOS) plots. Pole figure (PF) and inverse PF (IPF), were calculated and visualized using the MTEX package [32]. Micro-texture analysis was performed through the computation of orientation distribution functions (ODF) by the harmonic expansion method with the assumption of axial symmetry of an orthorhombic sample.

## 3. Results and Discussion

### 3.1. Macroscopic Evaluation

**Figure 1:**
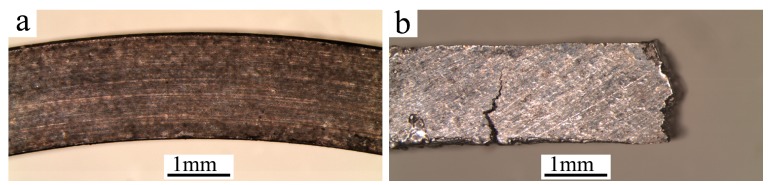
Macroscopic observation of (**a**) a ring at the initial state; and (**b**) a fragmented piece from a ring that was air-cooled after the expansion test.

Figure 1a,b illustrates a ring prior and post an expansion test, respectively. As a result of the expansion under high strain, the ring was fragmented into 8 pieces. Several events of plastic instability took place simultaneously along the expanded ring, with each event starting as a slight neck and most developing into a fracture. Figure 1b is one of the fragments that was air-cooled after the expansion test. In addition to a brittle fracture (right edge, Figure 1b), the fragmented piece also includes a crack, running transversely, from the inner diameter towards the outer diameter of the ring. Once the entire ring is expanded, the thickness of the ring is reduced. The expansion of the ring is associated with radial compression and circumferential tensile deformation.

### 3.2. Microscopic Characterization and Texture Analysis

**Figure 2:**
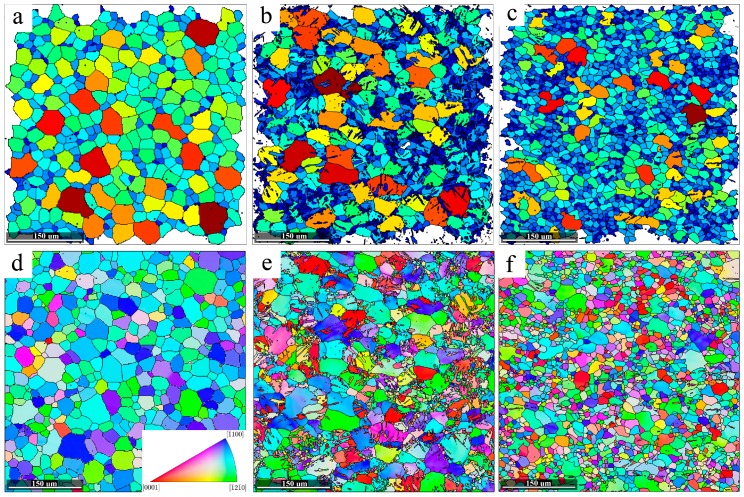
(**a**–**c**) grain-structure maps and (**d**–**f**) inverse pole figures along the extrusion direction (ED), from (**a**,**d**) the initial state; (**b**,**e**) the as-quenched specimens; and (**c**,**f**) the air-cooled specimens. In (**d**), an inset color-coded key triangle indicates crystallographic orientations.

EBSD ‘grain-structure maps’ from the initial, as-quenched and air-cooled specimens are presented in Figure 2a–c, respectively. These types of maps are based on grain-boundary misorientation criteria and grain-size measurements (defined in the experimental section). The grains are color-coded according to their size, from blue to red for fine and coarse grains, respectively. As can be seen in Figure 2a, the microstructure in the initial state consists of fine equiaxed grains, laced along slightly coarse-sized parent grains, as a consequence of recrystallization during extrusion. The average grain size of the finer grains is about 25 µm (averaged over 430 grains), and they occupy most of the investigated area (~80%). The average grain size of the coarse grains is about 52 µm (averaged over 20 grains). 

The microstructure of the as-quenched specimen comprises of coarse and fine grains, as in the initial state, yet the areal coverage, morphology, distribution and size of the fine grains are different (Figure 2b). In addition to fine equiaxed grains at the perimeter of the coarse grains, the microstructure also includes a large density of asymmetric fine grains with serrated grain boundaries. These grains are closely packed into colonies, an indication of dynamic recrystallization (DRX). The average grain size of the finer grains is about 6 µm (averaged over 2400 grains), and they occupy half of the investigated area. The average grain size of the coarse grains is about 50 µm (averaged over 60 grains). One of the most obvious features differentiating this microstructure from that of the initial state is the formation of large and broad deformation twins in the remains of the parent coarse grains. Propagation of twins in the parent grains is observed mainly from a grain boundary to the core, usually with lenticular-shaped morphology (as will be discussed later). Twins were not observed inside the fine equiaxed grains nor inside the asymmetric grains. 

In the air-cooled specimen, the areal fraction of densely packed fine grains decreased substantially, and the areal fraction of twins diminished almost completely (see Figure 2c). Instead, fine grains ripen into an equiaxed morphology with an average grain size of about 15 µm, occupying more than 80% of the investigated area. The major peak of the grain-size distribution (not shown) was shifted to a lower value. This outcome is a combination of the decreased areal fraction of the coarser grains (>20%) and their decreased averaged grain size (30 µm), which is close to that of the fine grains (20 µm). 

Further details on the evolution of the microstructure as a function of the expansion tests was obtained through the analysis of EBSD orientation maps, obtained from the ND–TD plane. Figure 2d–f present IPF maps along the ED for the initial state, the as-quenched and air-cooled specimens, respectively. The colors designate the crystallographic orientations, coded as shown in the inset color-key triangle (see Figure 2d). In the initial state, no simple correlation was found between the size of a grain and its orientation, as both fine and coarse grains contribute to a similar extent to the <$\bar{1}2\bar{1}0$>||ED and the <$\bar{1}100$>||ED components. It should be mentioned that these textural components represent most of the grains (~85% with max. deviation of 20°). Thus, the initial microstructure is characterized by a typical extrusion texture, also known as the basal texture, where most grains had their basal planes parallel to the ED or *c*-axes perpendicular to the ED. It should also be mentioned that this type of texture results in poor formability at room temperature [34]. Figure 2e presents the IPF along the ED for the as-quenched specimen. A color gradient was observed in coarse twin-free grains, both in the as-quenched and air-cooled specimen. Noting that the color of a grain represents its orientation, a color gradient implies a local disorientation in highly strained grains. It is plausible to assume that these grains consist of dense dislocations and subgrain boundaries. As the areal fraction of the coarse grains is smaller, the air-cooled specimen consists of a lower fraction of strained grains. In other words, the dynamic recovery progresses after fragmentation of the ring.

**Figure 3:**
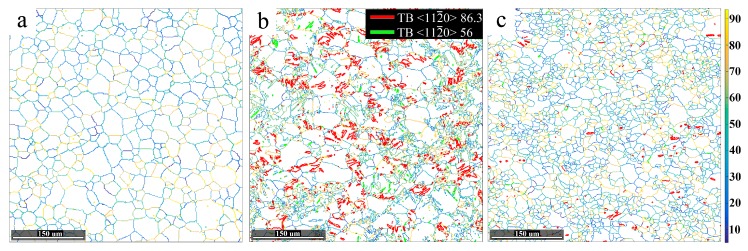

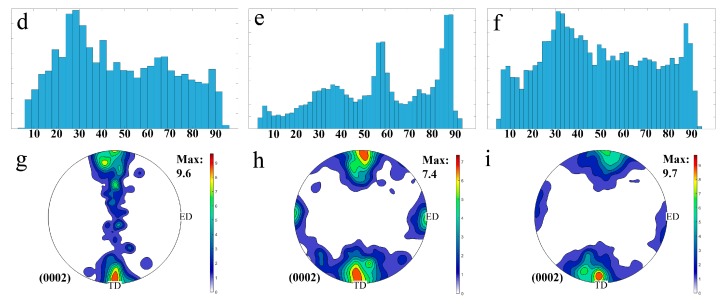
(**a**–**c**) Grain boundaries’ misorientation and twin boundaries (TBs); (**d**–**f**) misorientation angle distribution histograms and (**g**–**i**) (0002) pole figures (PF); from (**a**,**d**,**g**) the initial state, (**b**,**e**,**h**) the as-quenched and (**c**,**f**,**i**) the air-cooled specimens. Misorientation angles are marked based on the color-code bar right of Figure 3c. ED and TD, respectively, stand for the extrusion direction and transverse direction.

**Figure 4:**
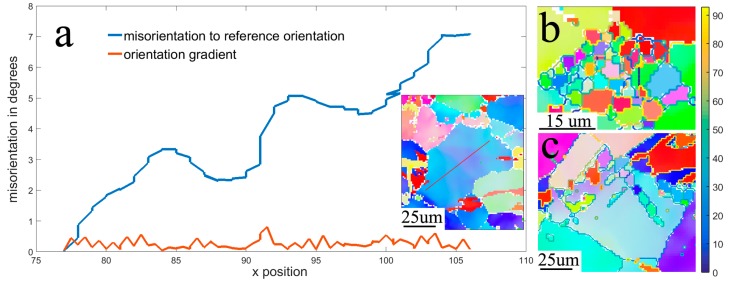
(**a**) a spatial profile extracted from a coarse grain presented in the inset to the right; (**b**) closely stacked fine grains in a coarse grain; (**c**) coarse grain that include fine grains nucleated from a fragmented twin.

Figure 3a–c display misorientation angle maps for the initial state, as-quenched and air-cooled specimens, respectively. Table 1 summarizes the results employing the misorientation angle criteria. For the as-quenched specimen, there is a drastic increase in the fraction of LAGBs while the fraction of HAGBs is substantially reduced, compared with the initial state. The microstructure of the as-quenched specimen consists of coarse grains which, according to the IPF map, present inner color variations (see Figure 2e). As an example, Figure 4a presents a segmented grain extracted from a ROI shown in Figure 2e. From that grain, a spatial profile was obtained. This profile was interpreted by means of the misorientation of each data point relative to the first one (Figure 4a, blue line) and by the misorientation of sequential data points (Figure 4a, red line). The intra-grain indicator suffers from high angular deviations, relative to the first one. This observation may be interpreted as the initial fragmentation of a coarse grain through the formation of dislocation walls as a mechanism for plastic accommodation. Small angular fluctuations (mostly, <1°) were observed for sequential data points (Figure 4a, red line). However, as the sensitivity of EBSD to misorientation variations is 0.5° to 2°, quantitative information in regards of dislocation density and inner boundaries cannot be obtained in a straightforward manner. The high density of closely packed fine grains also contributes to the drastic increase of the LAGBs fraction. An enlarged image of a colony of fine grains is presented in Figure 4b. Some of these grains are separated by LAGBs, formed by dislocation walls with small misorientation boundaries. As the expansion continues to failure, the fraction of HAGBs increases and resembles to that of the initial state. This increase can be attributed to two processes: (a) continuation of the subgrains’ rotation in order to maintain the infrastructure changes from LAGBs to HAGBs; (b) the higher mobility of LAGBs may result either in their elimination or coalescing.

**Figure 5:**
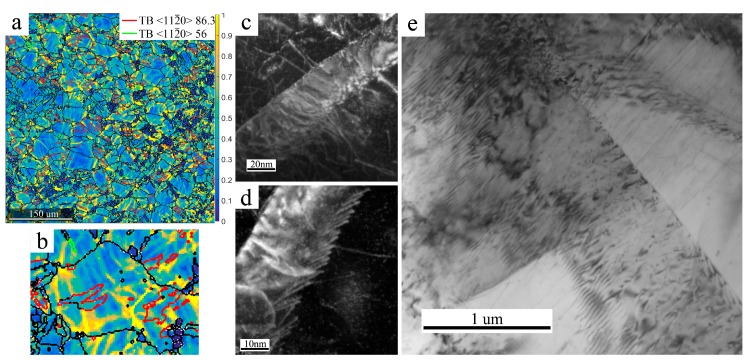
Twin analysis in the as-quenched specimen, based on (**a**) kernel-average misorientation (KAM) plot; In (**b**) is an enlarged region of interest (ROI) of a coarse grain containing a twin; (**c**–**e**) transmission-electron micrograph (TEM) near weak-beam dark-field (WBDF) conditions and conventional DF, showing dislocations threading the matrix and the twins, high density of dislocations within the twin lamella and dislocation network at the TBs.

In addition to the detailed boundary characterization described above, the extent of twinning was also estimated from the analysis of EBSD data. Interfaces satisfying the {$10\bar{1}2$} tensile twin boundaries (86° ± 5° <$11\bar{2}0$>) and {$10\bar{1}0$} compression twin boundaries (56° ± 5° <$11\bar{2}0$>) are marked in Figure 3a–c by red and green lines, respectively. Table 1 also describes the areal fraction of twins according to their type. There is a great deal of similarity between the microstructure of the post-expansion test to that of the initial state. In both, the areal fraction of twins is low to almost none. Consequently, their misorientation angle distribution histograms is similar, showing a diffused angular distribution (Figure 3d,f). In the as-quenched specimen, the EBSD analysis reveals a high density of twins in the coarse grains (see Figure 3b). Here, in contrast to the initial and air-cooled states, sharp peaks are noticed in the angular misorientation profile at 86° ± 5° and 56° ± 5°. As the majority of the twins are the tension-type, (see Figure 3e), their role in accommodation of the deformation is the most significant. Very low areal fraction of double compression-tension twins was also detected, and therefore not presented. Close examination of several twins shows that their boundaries are discontinues since they lose their 86° ± 5° relationship. The reason for that can be traced to the nucleation of recrystallized grains preferably at twin boundaries. Eventually, these grains consume the former twins, as can be seen in the enlarged image presented in Figure 4c. For a better understanding of this phenomenon, the strain incorporated in the as-quenched specimen was visualized using the kernel-average misorientation (KAM) map. As can be seen in Figure 5a,b, TBs environment are highly strained, as the local misorientation, or the average disorientation between each data point and its kernel is high. It is plausible to assume that large angular deviations in close proximity to a twin interface are consequences of a high density of dislocations in front of a twin interface. Further details on the TBs were obtained through TEM investigations. Figure 5c–e are TEM images recorded using near WBDF or conventional DF illumination. The high density of dislocations, tangled or in a network configuration, were observed in front of the TBs. The inner twins’ structure consists of dense dislocations, and dislocations threading the matrix and the twins. Thus, TBs act as barriers to the dislocation’s motion which is required for dynamic recovery.

In order to investigate the deformation mechanism in more detail, the (0002) pole figures (PFs) in the initial state, as-quenched and air-cooled specimens are shown in Figure 3g–i, respectively. The PF intensities are presented in multiples of random distribution (MRD). At the initial state, a typical extrusion texture with a characteristic fiber axis parallel to the ED is observed (*c*-axes perpendicular to the ED) [5]. The intense (0002) pole density is located parallel to the TD (Figure 3g). In the as-quenched specimen, the basal texture decreases (from 9.6 MRD to 7.4 MRD), with some rotations of *c*-axes from the orientation perpendicular to the ED towards orientation parallel to the ED (Figure 3h). The texture modification can be traced to several processes: the high-volume fraction of <$11\bar{2}0$> twins, which are activated during the expansion test, the subdivision of grains found inside the coarse ones, and the nucleation of randomly oriented, recrystallized grains. The obtained texture in the air-cooled specimen resembles to that shown for the initial state, with 9.7 MRD density of the (0002) parallel to the TD. Still, traces of the texture found in the as-quenched specimen are not completely eliminated (Figure 3i). This analysis is compatible with the results of the areal fraction of the twins prior and post expansion test (Table 1). One possible explanation for the strengthening of the basal texture in the air-cooled specimen is that it is a consequence of the mixed-mode deformation comprising twinning and slip. The TBs are a source of work hardening. It is plausible to assume that increased work hardening during the expansion test is suppressed by the activation of additional slip systems (prismatic and pyramidal) with higher critical resolved shear stress (CRSS) [5]. Activation of the prismatic slip is known to strengthen the basal texture [35]. 

### 3.3. A Proposal for a Modified Forming Technique

All previously described expanding-ring experiments ended in failure of the rings. Therefore, the forming of Mg-alloys employing the EMF technique in the conventional constellation has no apparent benefits. The applicability of this technique with its benefits was obtained by an experiment. In the modified expansion test, a ring was restrained by a mold. In this way, high elongation can be achieved, which is the major benefit of this method, without failure. As the electric current is not disrupted by the fragmentation of the ring, more complete annealing is expected.

**Figure 6:**
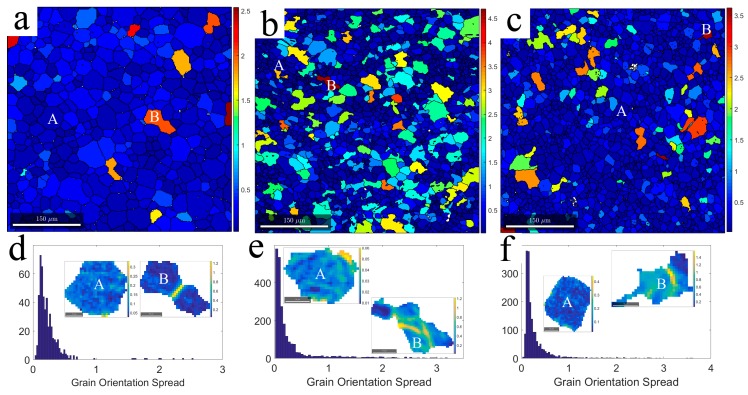
(**a**–**c**) grain-orientation spread (GOS) maps and (**d**–**f**) GOS distribution plots from (**a**,**d**) the initial state; (**b**,**e**) air-cooled; and (**c**,**f**) restrained air-cooled specimens. The color-code values are consistent with respective to GOS values, located on the right side of each map. Selected segmented grains (A, B) in each map are plotted using KAM analysis in the inset of each relevant GOS profile.

The ring restrained by a mold and air-cooled (non-quenched) is referred as a ‘restrained air-cooled’ specimen. Figure 6a–c present the GOS maps of the initial state, air-cooled and restrained air-cooled specimens, respectively. The GOS distribution plots of these samples are presented in Figure 6d–f. The value of GOS can be related with the degree of deformation. Analysis of these maps enables the new grains that were formed in the deformed matrix by the recrystallization process to be distinguished. From each map, two grains were segmented: A and B grains, presenting low and high GOS values, respectively. Using KAM analysis, their intra-grain structure was revealed (see Figure 6d–f). A consequence of the long homogenization treatment, the microstructure in the initial state exhibits a minor fraction of strained grains, corresponding to the low values in the GOS distribution histogram shown in Figure 6a,d, respectively. In the air-cooled specimen, highly deformed grains with GOS values even above 4.5° were observed along with grains with a low GOS value (<1°), Figure 6b,e. These values might be attributed to grains that underwent a dynamic recovery process. Compared to the air-cooled specimen, a lower fraction of strained grains is observed in the restrained air-cooled specimen. The GOS spread is narrower, with ranges between 0° and 3.5°. The fraction of grains with low GOS values observed in the restrained air-cooled specimen is much higher than that observed in the air-cooled specimen.

The GOS approach was also used to estimate the recrystallized grain fraction [36]. A GOS value of 0.75° was chosen as a cutoff for the orientation spread that distinguished between deformed and recrystallized grains. This threshold was set based on the analysis of the “fully recrystallized”, homogenized specimen. Further analysis of the recrystallization fraction was carried out using this GOS value for all specimens. As anticipated, the recrystallized fraction for the initial state and the restrained air-cooled were close (90% and 80%, respectively) while a lower value was calculated for the air-cooled sample (60%). Therefore, the modified expansion experiments combined enhanced elongation without failure with a high degree of dynamically annealed microstructure.

## 4. Summary

The result of large uniform elongation under high strain-rate deformation has been systematically investigated. The observed microstructural changes and textural modifications were facilitated by a mixed mode of deformation comprising activation of basal and non-basal slip systems, twinning and dynamic recrystallization. From the above analysis, the following processes take place during an EMF test: (1) deformation through active basal slip and nucleation of <$11\bar{2}0$> tension twins; (2) work hardening—dislocations’ entanglement at TBs and additional slip systems are activated; (3) nucleation of recrystallized fine grains at preferred sites such as inside coarse grains and at TBs; (4) growth of recrystallized grains which eventually consume twins.

Based on this work, an adopted formability technique is proposed. This modified version of the EMF expansion ring results in enhanced elongation without failure and a higher degree of dynamically annealed microstructure.

## Figures and Tables

**Table 1:** Grain-boundaries’ misorientation, area fraction of <$11\bar{2}0$> tension and compression twins.

Specimen	Grain Boundary Misorientation in [%]		Area Fraction of Twins in [%]	
	Low-Angle Grain Boundaries (LAGBs)	High-Angle Grain Boundaries (HAGBs)	<11–20> 86.3°	<11–20> 56°
Initial State	35	65	<0.5	<0.5
As-Quenched	77	23	14.5	2
Air-Cooled	37	63	4	<0.5
